# Supplementary material for: Lightweight data management with dtool
Source: PeerJ. 2019 Mar 7;7:e6562. doi: 10.7717/peerj.6562 (PMC6409086; doi:10.7717/peerj.6562)
Supplement: Supplemental Information 1 [file peerj-07-6562-s001.pdf]

Supplementary Material

Dataset creation

This paper makes use of three datasets hosted in Amazon S3.

- http://bit.ly/Ecoli-ref-genome
- http://bit.ly/Ecoli-reads
- http://bit.ly/Ecoli-reads-minified

Here are extended examples of how to create these datasets.

The scripts in this document are available at https://github.com/jic-dtool/dtool_examples.

Escherichia-coli-ref-genome

Create script (`create_reference_genome_dataset.sh`) for generating a reference genome dataset.

```
#!/usr/bash

# Usage  : ./create_reference_genome_dataset.sh <ACCESSION_ID>
# Example: ./create_reference_genome_dataset.sh U00096.3

# Exit immediately on failure of a command.
set -e

# Get the genome accession identifier from the command line.
ACCESSION_ID=$1

# Save working directory.
CUR_DIR=`pwd`

# Specify the URLs to use.
BASE_URL="https://www.ebi.ac.uk/ena/data/view/$ACCESSION_ID"
FASTA_URL="$BASE_URL&display=fasta"
TEXT_URL="$BASE_URL&display=text&header=true"

# Get the organism from the EMBL file format.
ORGANISM=`curl $TEXT_URL | grep '^OS' | sed s'/^OS   //'`


# Create the proto dataset.
```

```
DS_NAME=`echo $ORGANISM | cut -d" " -f1 -f2 | sed s'/ /-/'`-ref-genome
echo $DS_NAME
dtool create -q $DS_NAME

# Move into the data directory.
cd $DS_NAME/data

# Download the genome from the ENA.
FNAME=$ACCESSION_ID.fasta
curl $FASTA_URL > $FNAME

# Build the Bowtie2 indices.
INDEX_BUILDER=bowtie2-build
INDEX_BUILD_CMD="$INDEX_BUILDER $FNAME reference"
$INDEX_BUILD_CMD

# Move back to the original directory
cd $CUR_DIR

# Add descriptive metadata.
README=$DS_NAME/README.yml
echo "description: $ACCESSION_ID genome with Bowtie2 indices" > $README
echo "organism: $ORGANISM" >> $README
echo "accession_id: $ACCESSION_ID" >> $README
echo "link: $BASE_URL" >> $README
echo "index_builder: `$INDEX_BUILDER --version | head -1`" >> $README
echo "index_build_cmd: $INDEX_BUILD_CMD" >> $README

# Freeze the dataset.
dtool freeze $DS_NAME
```

Run script to create *E. coli* reference genome dataset.

```
$ bash create_reference_genome_dataset.sh U00096.3
```

Copy the dataset to Amazon S3 bucket.

```
$ dtool cp -q scripts/Escherichia-coli-ref-genome s3://dtool-demo/
s3://dtool-demo/8ecd8e05-558a-48e2-b563-0c9ea273e71e
```

Publish the dataset to make it world readable via HTTP.

```
$ dtool publish -q  \
   s3://dtool-demo/8ecd8e05-558a-48e2-b563-0c9ea273e71e
https://dtool-demo.s3.amazonaws.com/8ecd8e05-558a-48e2-b563-0c9ea273e71e
```

Create shortened URL for accessing the dataset: http://bit.ly/Ecoli-ref-genome

Escherichia-coli-reads-ERR022075

Create script (`create_ecoli_reads_dataset.sh`) for generating the dataset.

```
#!/bin/bash

# Exit immediately on failure of a command.
set -e

# Specify the dataset name and data directory.
DS_NAME=Escherichia-coli-reads-ERR022075
DATA_DIR=$DS_NAME/data

# Create an open proto dataset.
dtool create -q $DS_NAME

# Add descriptive metadata to to proto dataset.
cat <<'EOF' | dtool readme write $DS_NAME -
---
description: Whole Genome Sequencing of Escherichia coli str. K-12 MG1655
design: Paired-end sequencing (2x100 base) of E. coli library
sample: E. coli K-12 strain MG1655
study: |
  Paired-end sequencing of the genome of Escherichia coli K-12 strain MG1655
  using the Illumina Genome Analyzer IIx
Library:
  Name: CT1093
  Instrument: Illumina Genome Analyzer IIx
  Strategy: WGS
  Source: GENOMIC
  Selection: RANDOM
  Layout: PAIRED
  Construction protocol: |
    Standard Illumina paired-end library construction protocol. Genomic DNA was
    randomly fragmented using nebulisation and a ~600 bp fraction (including
    adapters) was obtained by gel electrophoresis.
links:
  - SRA: https://www.ncbi.nlm.nih.gov/sra/ERX008638
  - ENA: https://www.ebi.ac.uk/ena/data/view/ERX008638
EOF

# Add data to to proto dataset.
wget --directory-prefix $DATA_DIR  \
   ftp://ftp.sra.ebi.ac.uk/vol1/fastq/ERR022/ERR022075/ERR022075_1.fastq.gz
wget --directory-prefix $DATA_DIR  \
   ftp://ftp.sra.ebi.ac.uk/vol1/fastq/ERR022/ERR022075/ERR022075_2.fastq.gz
```

```
# Convert the proto dataset into a dataset by freezing it.
dtool freeze $DS_NAME
```

Run script to create *E. coli* reads dataset.

```
$ bash create_ecoli_reads_dataset.sh
```

Copy the dataset to Amazon S3 bucket.

```
$ dtool cp -q Escherichia-coli-reads-ERR022075 s3://dtool-demo/
s3://dtool-demo/faa44606-cb86-4877-b9ea-643a3777e021
```

Publish the dataset to make it world readable via HTTP.

```
$ dtool publish -q  \
   s3://dtool-demo/faa44606-cb86-4877-b9ea-643a3777e021
https://dtool-demo.s3.amazonaws.com/faa44606-cb86-4877-b9ea-643a3777e021
```

Create shortened URL for accessing the dataset: http://bit.ly/Ecoli-reads

Escherichia-coli-reads-ERR022075-minified

Create script (`minify.sh`) for taking an input dataset and extracting the first 4000 lines from each item and putting these into a minfied version of the dataset.

```
#!/bin/bash

# Exit immediately on failure of a command.
set -e

# Read in the input from the command line.
INPUT_URI=$1
OUTPUT_BASE_URI=$2
NUM_LINES=4000

# Create a name for the output dataset based on the input dataset.
OUTPUT_NAME=`dtool name $INPUT_URI`-minified

# Create an open proto dataset.
OUTPUT_URI=`dtool create -q $OUTPUT_NAME $OUTPUT_BASE_URI`

# Process all the items in the input dataset and
```

```
# add the results to the output dataset.
for ITEM_ID in `dtool identifiers $INPUT_URI`; do

  # Fetch the item from the dataset and get an absolute path
  # from where its content can be accessed.
  ITEM_ABSPATH=`dtool item fetch $INPUT_URI $ITEM_ID`

  # Write the minified version of the item to a temporary file.
  TMP_MINIFIED=$(mktemp /tmp/minfied.XXXXXX)
  gunzip -c $ITEM_ABSPATH | head -n $NUM_LINES | gzip > $TMP_MINIFIED

  # Add the temporary file to the output dataset giving it the relpath
  # of the item from the input dataset.
  RELPATH=`dtool item relpath $INPUT_URI $ITEM_ID`
  dtool add item $TMP_MINIFIED $OUTPUT_URI $RELPATH

  # Cleanup.
  rm $TMP_MINIFIED
done

# Create descriptive metadata for the output dataset.
TMP_README=$(mktemp /tmp/dtool-readme.XXXXXX)
dtool readme show $INPUT_URI > $TMP_README
echo "minified:" >> $TMP_README
echo "  from_UUID: `dtool uuid $INPUT_URI`" >> $TMP_README
echo "  from_URI: $INPUT_URI" >> $TMP_README
echo "  content: first $NUM_LINES per item" >> $TMP_README

# Add the descriptive metadata to the output dataset.
dtool readme write $OUTPUT_URI $TMP_README

# Cleanup.
rm $TMP_README

# Finalise the output dataset.
dtool freeze $OUTPUT_URI
```

Run script to create the minified *E. coli* reads dataset.

```
$ bash minify.sh s3://dtool-demo/faa44606-cb86-4877-b9ea-643a3777e021  \
   s3://dtool-demo
Dataset frozen s3://dtool-demo/907e1b52-d649-476a-b0bc-643ef769a7d9
```

Publish the dataset to make it world readable via HTTP.

```
$ dtool publish -q  \
   s3://dtool-demo/907e1b52-d649-476a-b0bc-643ef769a7d9
https://dtool-demo.s3.amazonaws.com/907e1b52-d649-476a-b0bc-643ef769a7d9
```

Create shortened URL for accessing the dataset: http://bit.ly/Ecoli-reads-minified

Creating a directory with the sample datasets

To try out the `dtool ls` and `dtool inventory` commands it is useful to have a directory containing sample datasets. Below are the commands required to create such a directory.

```
$ mkdir my_datasets
$ dtool cp http://bit.ly/Ecoli-ref-genome my_datasets
$ dtool cp http://bit.ly/Ecoli-reads my_datasets
$ dtool cp http://bit.ly/Ecoli-reads-minified my_datasets
```

Bowtie2 processing example

Aligning paired reads with Bowtie2 is more involved than the `minify.sh` example as the command invocation requires a pair of dataset items. This can be achieved by making use of per item metadata. Specifically, by having per item metadata specifying if an item refers to a forward or a backward read and another per item metadata specifying the identifier of the paired read. Below is a Python script (`create_paired_read_overlays_from_fname.py`) for creating such per item metadata as so called "overlays" to a dataset. The `is_read1` overlay is set to "true" for forward reads and "false" for backward reads. The `pair_id` overlay is used to lookup the item identifier of the paired read. The script also creates an overlay named `useful_name` that is useful for creating meaningful names for the output of the alignment.

```
import os

import click

from dtool_cli.cli import dataset_uri_argument
from dtoolcore import DataSet


def is_file_extension_in_list(filename, extension_list):
    for extension in extension_list:
        if filename.endswith(extension):
```

```
            return True

    return False


def identifier_and_read_from_relpath(relpath):
    fname = os.path.basename(relpath)  # ERR188234_chrX_2.fq.gz
    name = fname.split(".")[0]  # ERR188234_chrX_2
    name_parts = name.split("_")  # ERR188234, chrX, 2
    return "_".join(name_parts[:-1]), int(name_parts[-1])


def create_read1_overlay(dataset):

    read1_overlay = {}

    for i in dataset.identifiers:
        relpath = dataset.item_properties(i)['relpath']

        if not is_file_extension_in_list(
            relpath,
            ['fq', 'fq.gz', 'fastq', 'fastq.gz']
        ):
            continue

        identifier, read = identifier_and_read_from_relpath(relpath)
        if read == 1:
            read1_overlay[i] = True

    for i in dataset.identifiers:
        if i not in read1_overlay:
            read1_overlay[i] = False

    dataset.put_overlay('is_read1', read1_overlay)


def create_pair_id_overlay(dataset):

    data = {}
    for i in dataset.identifiers:
        relpath = dataset.item_properties(i)['relpath']

        if not is_file_extension_in_list(
            relpath,
            ['fq', 'fq.gz', 'fastq', 'fastq.gz']
        ):
```

```
            continue

        identifier, read = identifier_and_read_from_relpath(relpath)

        item = data.get(identifier, {})
        item[read] = i

        data[identifier] = item

    pair_id = {}
    for item in data.values():
        one, two = item.values()
        pair_id[one] = two
        pair_id[two] = one

    for i in dataset.identifiers:
        if i not in pair_id:
            pair_id[i] = None

    dataset.put_overlay('pair_id', pair_id)


def create_useful_name_overlay(dataset):

    useful_name_overlay = {}

    for i in dataset.identifiers:
        relpath = dataset.item_properties(i)['relpath']

        if not is_file_extension_in_list(
            relpath,
            ['fq', 'fq.gz', 'fastq', 'fastq.gz']
        ):
            continue

        identifier, read = identifier_and_read_from_relpath(relpath)
        useful_name_overlay[i] = identifier

    for i in dataset.identifiers:
        if i not in useful_name_overlay:
            useful_name_overlay[i] = False

    dataset.put_overlay('useful_name', useful_name_overlay)


@click.command()
```

```
@dataset_uri_argument
def cli(dataset_uri):

    dataset = DataSet.from_uri(dataset_uri)
    create_read1_overlay(dataset)
    create_pair_id_overlay(dataset)
    create_useful_name_overlay(dataset)


if __name__ == '__main__':
    cli()
```

This script was applied to the Escherichia-coli-reads-ERR022075 and Escherichia-coli-reads-ERR022075-minified datasets.

```
$ python create_paired_read_overlays_from_fname.py  \
   s3://dtool-demo/faa44606-cb86-4877-b9ea-643a3777e021
$ python create_paired_read_overlays_from_fname.py  \
   s3://dtool-demo/907e1b52-d649-476a-b0bc-643ef769a7d9
```

It is possible to list and view overlays using the `dtool overlay ls` and `dtool overlay show` commands.

```
$ dtool overlay ls http://bit.ly/Ecoli-reads
pair_id
is_read1
useful_name
$ dtool overlay show http://bit.ly/Ecoli-reads is_read1
{
  "9760280dc6313d3bb598fa03c5931a7f037d7ffc": true,
  "8bda245a8cd526673aab775f90206c8b67d196af": false
}
```

It is also possible to retrieve the value of an overlay for a specific item using the `dtool item overlay` command.

```
$ dtool item overlay pair_id http://bit.ly/Ecoli-reads  \
   9760280dc6313d3bb598fa03c5931a7f037d7ffc
8bda245a8cd526673aab775f90206c8b67d196af
```

The script below (`bowtie2_align.sh`) uses dataset to dataset processing to align reads to a reference genome.

```
#!/bin/bash

# Exit immediately on failure of a command.
set -e

# Read in the input from the command line.
READS_URI=$1
REF_GENOME_URI=$2
OUTPUT_BASE_URI=$3

# Specify the command for performing the alignment.
CMD=bowtie2

REF_DIR=`dtool name $REF_GENOME_URI`
REF_DATA_DIR=$REF_DIR/data
REF_DATA_PREFIX=$REF_DIR/data/reference

if [ ! -d $REF_DIR ]; then
    # The dataset has not been copied yet.
    dtool cp $REF_GENOME_URI .
fi

# Specify the name of the output dataset and create it.
OUTPUT_NAME=`dtool name $READS_URI`-bowtie2-align
OUTPUT_URI=`dtool create -q $OUTPUT_NAME $OUTPUT_BASE_URI`


# Perform the alignment and add the output file to the output dataset.
for ITEM_ID in `dtool identifiers $READS_URI`; do
  if [ `dtool item overlay is_read1 $READS_URI $ITEM_ID` = "True" ]; then
     PAIR_ID=`dtool item overlay pair_id $READS_URI $ITEM_ID`
     READ1_ABSPATH=`dtool item fetch $READS_URI $ITEM_ID`
     READ2_ABSPATH=`dtool item fetch $READS_URI $PAIR_ID`
     TMP_SAM=$(mktemp /tmp/sam_output.XXXXXX)
     $CMD -x $REF_DATA_PREFIX -1 $READ1_ABSPATH -2 $READ2_ABSPATH -S $TMP_SAM
     SAM_NAME=`dtool item overlay useful_name $READS_URI $ITEM_ID`.sam
     dtool add item $TMP_SAM $OUTPUT_URI $SAM_NAME
     rm $TMP_SAM
  fi
done

# Create descriptive metadata for the output dataset.
TMP_README=$(mktemp /tmp/dtool-readme.XXXXXX)
echo "description: bowtie2 alignment" > $TMP_README
echo "input_reads_uri: $READS_URI" >> $TMP_README
echo "ref_genome_uri: $REF_GENOME_URI" >> $TMP_README
```

```
echo "bowtie_version: `$CMD --version | head -1`" >> $TMP_README
cat $TMP_README

# Add the descriptive metadata to the output dataset
dtool readme write $OUTPUT_URI $TMP_README

rm $TMP_README

# Finalise the output dataset.
dtool freeze $OUTPUT_URI
```
